# Supplementary material for: Selection of forage grasses for cultivation under water-limited conditions using Manhattan distance and TOPSIS
Source: PLoS One. 2024 Jan 2;19(1):e0292076. doi: 10.1371/journal.pone.0292076 (PMC10760912; doi:10.1371/journal.pone.0292076)
Supplement: Attachment — Submitted filename: Response to Reviewers.docx [file pone.0292076.s002.docx]

**Response to reviewers**

Below we list the reviewers' comments and the actions taken by the authors to adapt the text to each of the considerations, with the consequent justifications for the actions taken.

| **Reviewer #1** | |
| --- | --- |
| **Comment** | **Action** |
| Row # 74 – 77: You gave information concerning drought tolerance only to 2 species used in experiment. But what about drought relations known from literature concerning other grass species used? | In this description we focused only on those grasses that were employed in previous works with responses to water stress or drought. |
| Row # 90 – 91: It should be stated more general - not only to tropical forage grasses. The understanding of plant reaction to water stress is crucial to further reading,... | Has been corrected in the text |
| Row # 96 – 97: Multivariate analysis methods are widely used in other disciplines, not only agriculture. | The text has been changed to “Multivariate analysis methods are employed in many areas, including agriculture, where they can help to choose the most robust species of tropical forage grasses when under stress conditions, for example.” |
| Row # 131: Give detail information concerning: seed sowing amount (g/pot or g/square m), date of sowing, any treatment applied after sowing (i.e. seed covering, watering etc.) before seedling emerged. You should also add information concerning experiment design, i.e. number of pots per cultivar, number of observations per pot etc. | Has been corrected in the text |
| Row # 148 – 149: Where do you know from that this PC values were 'moderate drought' or 'severe drought'? | Has been corrected in the text |
| Row # 149 - 150: Yes, but this is only one example of possible drought - plant development relation. Drought may come also in other plant development stages. Are the effect of drought exactly during 25 days at tillering and stalk elongation significantly critical for further plant development? What was the idea of choosing exactly this time of grass development? | The production of an adequate amount of straw in the e no-tillage system has been difficult for the Brazilian Cerrado region because of inadequate soil water availability during the region's dry season. The volume and frequency of rainfall in the Cerrado fluctuate and are insufficient, especially during the fall, winter and early spring, thus reducing the chances of adequate water supply for the crops. After harvesting the main crop (generally soybeans), cover crops are sown that may be subject to lack of water (drought stress). Generally, there is some precipitation that allows the seeds to germinate, but during tillering there is drought stress. Hence the idea of choosing drought stress for exactly 25 days at tillering. |
| Row # 163: Please, add correct citation to the references of the Kuijper’s leaf numbering system. | has been corrected in the text |
| Row # 163 – 164: It is necessary to know how many live seeds were sown per pot. | has been corrected in the text #134-136 |
| Row # 295 – 298: Figures are very good but it lacks significance of difference between means. Therefore in some case there is no effect of the drought conditions on trait mean values. I do suggest to add some ANOVA tables to express the effect of treatments applied. | The ANOVA Table has been added. Table 3. |
|  |  |
| **Reviewer #2** | |
| **Comment** | **Action** |
| 1. Overall manuscript needs English improvement. | It was corrected after a rigorous reading and with an English corrector. |
| 2. There is no molecular based evidence to highlight the reasons the better variety in water deficit stress. I suggset, molecular based research may highlight more on this way. | A revision was made to the text (lines 82 to 84). |
| 3. References should be rechecked and arrange according to the journal instruction | All references have been numbered in order of citation and their position in the references section has been corrected. |
| 4. No author Contributions mentioned in manuscript. | The contributions of each of the authors has been added and also supporting information |
| 5. No innovated research found in this research treatment. | Although the treatment itself is not innovative, the method used for selection is innovative. The method that uses the Manhattan distance and TOPSIS has never been used before for selecting the genotypes researched in the presented work. Therefore, the innovative feature resides in the application of this new methodology, which is worth noting, was proposed by the authors of the work in a recently paper. |
| Keep in word limits to 300 in abstract section. | The number of words was reduced to 298. |
| Line 55: Use appropriate keywords please. | Appropriate keywords were inserted. |
| Line 59: Please recheck the report year and the figures written? 2023 or 2019? | The reference has been updated |
| Line 79: delete off-season. | Done |
| Line 103: Rewrite this line short and more understandable. | The text has been improved |
| Line 107: Forage plants | The text has changed since the previous change |
| Introduction portion is very lengthy. Please make it short and write essential about problem that can make more easily understandable to the irrelevant reader of your major subject. | Introduction section has been shortened and rewritten for better comprehension. |
| Line 202: Make it short please. | It is not possible, because it is the name of the method. |
| No statistical analysis of the values or parameters that taken from the plants. | The following text was added to the end of the "Quantification of morphological traits" section: *"Since the objective of the present study is to employ the selection method proposed by [16], the statistical analyzes of the experiments are not displayed here. They can be consulted in [7]"* |
| Line 341: delete “it”. | Done |
| Line 361: Put space between above ground. | Done |
| Line 363: Put comma next to side. | Done |
| Line 364: Put comma next to sections. | Done |
| Line 369: Shift table 2 at the end of the sentence, as shown in Table 2. | Done |
| Line 406: Put comma next to comparing. | Done |
|  |  |
| **Reviewer #3** | |
| **Comment** | **Action** |
| 1. Cite other studies that have used TOPSIS in the evaluation of plant varieties or genotypic selection. | We've added three references to the end of the "Technique for Order Preference by Similarity to Ideal Solution (TOPSIS)" section |
| 2. Before showing normalized values in control and stressed conditions, please include a table to show your raw data of the morphological characteristics of 9 grass cultivars subjected to control and drought stress conditions. | Table 1 with the raw data was added |
| 3. As mentioned in the introduction, the data in this paper was obtained in the experiment carried out by Zuffo et al. (2022) using nine cultivars from five grass species. Zuffo et al. (2022) used canonical correlation to analyze drought indices as compared to this research which used Manhattan and TOPSIS to analyze morphological variables. However, using the same data, the study of Zuffo et al. (2022) found that Panicum maximum ‘Mombaca’ has greater adaptability and stability under water stress while your study identified Pennisetum glaucum ‘ADR. 300’, Paspalum atratum ‘Pojuca’, Urochloa brizantha ‘Marandu’ and ‘Xaraés’ as the best varieties with greater adaptability and stability. As such, there are 2 contrasting results using the same data. Discuss further your varying results in the discussion (L353–363)? | Texts have been modified and explanations added (rows 396-412). |
| 4. Could you explain what “greater adaptability and stable drought tolerance mean”? Why is ‘Xaraés’  which has a TOPSIS score of 0.6 included among other cultivars with greater adaptability and stable  drought tolerance? | The text was corrected so that it only had the word 'stability'. In this case, stability means that there was less change in the stress environment compared to the control environment. |
| 5. The authors alternatively used variety or cultivar in the manuscript. Since these are established  grasses, it is better to use “cultivar” and maintain consistency in the manuscript. | The word "variety" has been replaced by "cultivate" throughout the text. |
| Abstract:  Use single apostrophes, not double, for cultivar names here and elsewhere in the manuscript. (eg.,  ‘Pojuca’, instead of “Pojuca”).  “Therefore”, instead of “therefore”, in second to the last sentence. | Done |
| L63 Replace “(Feltran-Barbieri et al., 2021)” with “(Feltran-Barbieri & Féres, 2021)”. | All citations have been changed to the required standard as requested by Reviewer #2 |
| L73, 85, 90 Replace “Mastalerczuk et al., 2021” with “Mastalerczuk & Borawska-Jarmułowicz, 2021”. | All citations have been changed to the required standard as requested by Reviewer #2 |
| L104 What do you mean with “forage grasses tree genotypes”? | This sentence has since been changed, due to Reviewer #2 comment |
| L107–108 Restate the incomplete sentence “forage plants resistant to stress conditions”. | This sentence has since been changed, due to Reviewer #2 comment |
| L140–144 Use en dash, not hyphen, to indicate a negative value (eg. Kg–1 for Kg-1) | Done |
| L164 NL or NGL? | Occurrences of "NL" have been corrected for "NGL" |
| L187 Replace “Aggarwal, Hinneburg, and Keim” with “Aggarwal et al.” | All citations have been changed to the required standard as requested by Reviewer #2 |
| L217 Replace “Tzeng, 2011” with “Tzeng & Huang, 2011”. | All citations have been changed to the required standard as requested by Reviewer #2 |
| L218 Replace “Yadav, 2019” with “Yadav et al., 2019”. | All citations have been changed to the required standard as requested by Reviewer #2 |
| L339 Replace “Tzeng, 2011” with “Tzeng & Huang, 2011”. | All citations have been changed to the required standard as requested by Reviewer #2 |
| L341 Complete the sentence for “The TOPSIS method”. | The entire paragraph has been corrected. |
| L348 Replace “(Aggarwal, Hinneburg, and Keim, 2001)” with “(Aggarwal et al., 2002)”. | All citations have been changed to the required standard as requested by Reviewer #2 |
| L354–363 When writing cultivar names, “cv.” is replaced with single apostrophes on cultivar names (eg.  Pennisetum glaucum ‘ADR 300’, instead of Pennisetum glaucum cv. “ADR 300”). Please use single apostrophes consistently for all cultivar names here and elsewhere in the manuscript. | Done |
| L357 Delete the comma (,) after “others”. | This sentence has since been changed, due to Reviewer #2 comment |
| L359 Replace “Zufo et al” with “Zuffo et al.”. | All citations have been changed to the required standard as requested by Reviewer #2 |
| L359–363 The phrase “a similar forage species to ADR300” which is referring to Panicum maximum ‘Mombaca’ is obviously redundant and may cause confusion as to what it implies. Please delete or modify this phrase. | The sentence has been deleted |
| L362 ‘ADR 300’, not ADR30 | The expression has been corrected |
| L389 “choosing analyzing”? | The sentence has been changed to “As we are in an eight-dimensional space, it is not possible to analyze all variables simultaneously.” |
| L391–393 Revise the sentence for clarity. | Done |
| L411–412 Use italics for scientific names and single apostrophe for cultivar names | The sentence has been changed to “Comparing the four worst varieties, according to the results presented in Figure 3 and Table 4, it is not so clear why the 'Tanzânia' cultivar is the worst according to TOPSIS. However, when we analyze the percentage variation signs, this selection remains evident. Although in absolute values the 'Tanzânia' cultivar changed less than the 'BRS Piatã' cultivar, the former had a negative variation. This makes the calculated distance much larger. It is impossible to visualize this in a space of size greater than 3. But this is the expected result when you have a vector whose modulus is greater than the others. That is, in this case, the value of the NT variable in the moderate water stress environment is greater than in the control environment. On the other hand, the other variables have a smaller measure. This results in a vector with much greater length. The same can be observed for the other varieties.” |
| References:  Follow the journal’s style and format.  Is there a period after doi?  Use an en dash (–), not a hyphen (-), when writing page ranges.  L463–465 The authors were not cited in the manuscript. | All references have been changed to the required standard as requested by Reviewer #2 |
